# Simple Sequence Repeats Together with Mismatch Repair Deficiency Can Bias Mutagenic Pathways in *Pseudomonas aeruginosa* during Chronic Lung Infection

**DOI:** 10.1371/journal.pone.0080514

**Published:** 2013-11-21

**Authors:** Alejandro J. Moyano, Sofía Feliziani, Julio A. Di Rienzo, Andrea M. Smania

**Affiliations:** 1 Centro de Investigaciones en Química Biológica de Córdoba (CIQUIBIC), CONICET, Departamento de Química Biológica, Facultad de Ciencias Químicas, Universidad Nacional de Córdoba, Córdoba, Argentina; 2 Estadística y Biometría, Facultad de Ciencias Agropecuarias, Universidad Nacional de Córdoba, Córdoba, Argentina; Universidad Nacional de La Plata, Argentina

## Abstract

*Pseudomonas aeruginosa* is an opportunistic pathogen that chronically infects the airways of cystic fibrosis (CF) patients and undergoes a process of genetic adaptation based on mutagenesis. We evaluated the role of mononucleotide G:C and A:T simple sequence repeats (SSRs) in this adaptive process. An *in silico* survey of the genome sequences of 7 *P. aeruginosa* strains showed that mononucleotide G:C SSRs but not A:T SSRs were greatly under-represented in coding regions, suggesting a strong counterselection process for G:C SSRs with lengths >5 bp but not for A:T SSRs. A meta-analysis of published whole genome sequence data for a *P. aeruginosa* strain from a CF patient with chronic airway infection showed that G:C SSRs but not A:T SSRs were frequently mutated during the infection process through the insertion or deletion of one or more SSR subunits. The mutation tendency of G:C SSRs was length-dependent and increased exponentially as a function of SSR length. When this strain naturally became a stable Mismatch Repair System (MRS)-deficient mutator, the degree of increase of G:C SSRs mutations (5-fold) was much higher than that of other types of mutation (2.2-fold or less). Sequence analysis of several mutated genes reported for two different collections, both containing mutator and non-mutator strains of *P. aeruginosa* from CF chronic infections, showed that the proportion of G:C SSR mutations was significantly higher in mutators than in non-mutators, whereas no such difference was observed for A:T SSR mutations. Our findings, taken together, provide genome-scale evidences that under a MRS-deficient background, long G:C SSRs are able to stochastically bias mutagenic pathways by making the genes in which they are harbored more prone to mutation. The combination of MRS deficiency and virulence-related genes that contain long G:C SSRs is therefore a matter of concern in *P. aeruginosa* CF chronic infection.

## Introduction

*Pseudomonas aeruginosa* is an opportunistic pathogen that chronically infects the lungs and airways of cystic fibrosis (CF) patients and persists by undergoing a genetic adaptation based on mutagenic processes [1]. Mononucleotide simple sequence repeats (SSRs) are known to function as mutagenic hotspots within genes or gene regulatory regions [2], [3] and may therefore play an important role in this mutagenic adaptive process.

The mutagenic properties of mononucleotide SSRs have been studied in several individual genes and found to vary among species. Long mononucleotide SSRs are under-represented in the genomes of bacteria and show signs of strong counterselection at lengths greater than 7-8 bp [4], [5]. In an *in vitro* study of the factors involved in the mutagenic process of a specific gene, we demonstrated recently that a Mismatch Repair System (MRS)-deficient mutator strain of *P. aeruginosa* (this type of mutator is typically found in CF chronic infections [6]) differed in its mutagenic spectrum from its wild-type counterpart and that mutations are biased toward mononucleotide G:C SSRs under this mutator background and are therefore hotspots for mutagenesis [7], [8]. No study to date has attempted to analyze simultaneously the functional relevance of all the SSRs of a specific organism during the process of genetic adaptation to a particular environment. There have also been no genome-scale studies on SSRs in *P. aeruginosa*.

We initially used publicly available software tools to analyze the frequency and distribution of mononucleotide G:C and A:T SSRs in the coding and non-coding genome regions of 7 *P. aeruginosa* strains whose genome sequences and annotations are available online. We then investigated the role of these mononucleotide SSRs in the pathogenic adaptive process of *P. aeruginosa* to the chronic lung infection environment of CF patients. For this purpose, we performed a meta-analysis on published whole-genome sequence data from a single *P. aeruginosa* strain that had been sequentially isolated from the airways of a CF patient during a 90-month period [1]. This strain eventually became a MRS-deficient mutator, a condition that may enhance the genetic adaptation to the airways of CF patients [9]. We analyzed separately the roles of G:C and A:T SSRs before and after this strain attained the mutator state.

Our findings suggest that (i) G:C SSRs but not A:T SSRs show signs of a counterselection process in the genomic coding regions of *P. aeruginosa* and (ii) under a MRS-deficient mutator background, the G:C SSRs constitute an important pathway in the mutation-based adaptation of *P. aeruginosa* to the chronic lung infection environment of CF patients.

## Results and Discussion

### Mononucleotide SSRs in the genome of *P. aeruginosa*: G:C but not A:T SSRs show signs of a counterselection process

**Figure 1:**
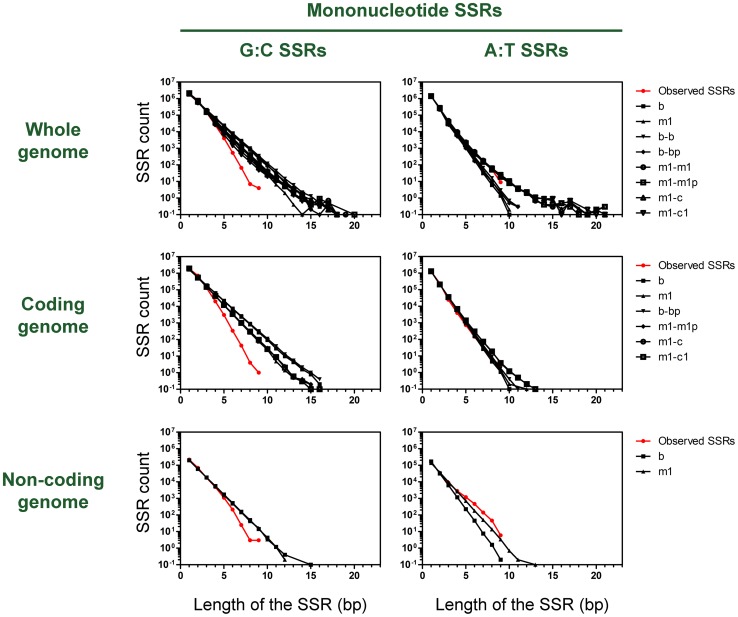
Mononucleotide SSRs in the *P. aeruginosa* PAO1 genome. The plots show the counts for mononucleotide SSRs (red circles) in the whole *P. aeruginosa* PAO1 genome and in random sequences generated by various predictive models (black symbols). b and m1: homogeneous models (Bernoulli and first-order Markov); b-b, b-bp, m1-m1, m1-m1p, m1-c and m1-c1: heterogeneous models (see Methods). Counts are shown of mononucleotide G:C and A:T SSRs in the coding and non-coding regions of the genome.

To clarify the role of mononucleotide SSRs in the genetic adaptation of *P. aeruginosa*, we first evaluated the SSR content in the genomic sequences of 7 *P. aeruginosa* strains. These genomes varied in size from 6.2 to 6.9 Mb and had a G:C content of ∼67%. Assuming that nucleotides are randomly distributed and that homopolymeric SSRs are neutral objects along the genome, their frequencies should be similar to those obtained from random predictive models. Based on this assumption, we performed an *in silico* genome-wide search of homopolymeric SSRs and compared the observed frequencies of mononucleotide SSRs with the values obtained from 8 previously described random predictive models [10]. The results for strains PAO1 and PACS2 are shown in Figure 1 and S1, respectively. The results for strains PA14, LESB58, 2192, 39016, and PA7 were similar (data not shown). The observed frequencies of short homopolymeric SSRs (length ≤4 bp) did not differ from the expected values of a random distribution, whereas longer SSRs were clearly under-represented in the 7 analyzed genomes and SSRs with lengths ≥9 bp were absent. By distinguishing between G:C and A:T SSRs, we found that the under-representation of long homopolymeric SSRs was attributable exclusively to the G:C SSRs and that the frequencies of G:C SSRs with lengths >5 bp were significantly lower than the expected values of a random distribution (Figure 1 and S1). This finding suggests that G:C SSRs have great intrinsic replicative instability and/or are subjected to a strong counterselective pressure, as has been observed for other bacterial species [4]. In regard to A:T SSRs, our analysis showed no under-representation even for SSRs with lengths up to 9 bp, and the frequencies were consistent with those from the random predictive models. This latter finding could have resulted from an over-representation of A:T SSRs in intergenic regions that masked a possible under-representation in coding DNA. To evaluate the role of selection in the observed representation of G:C and A:T SSRs, we evaluated the content of G:C SSRs in the coding regions (in which most selection occurs) and non-coding regions of the genome, and compared these values with the expected values from random models for both regions. If the observed under-representation resulted mainly from genetic instability during DNA replication, then we would expect to observe similar contents of G:C SSRs in the coding and non-coding regions. In fact, under-representation of G:C SSRs with lengths >4 bp was prominent in the coding regions but much less so in the non-coding regions (Figure 1 and S1) [4]. In regard to A:T SSRs, previous studies of other bacteria, such as *Escherichia coli* and *Mycobacterium tuberculosis*, showed that the above phenomenon was much less prominent than for G:C SSRs because the A:T SSRs had greater stability and lower mutation rates in comparison to the G:C SSRs, and clear under-representation was observed only for SSRs with lengths >6 bp [4], [11]. The high overlap between the number of intragenic A:T SSRs that we observed and the numbers predicted by the random models is interesting because it suggests a lack of selection against the occurrence of A:T SSRs within the genome coding regions of *P. aeruginosa*.

Our genome-level approach using an *in silico* genome-wide survey of mononucleotide SSRs is the first exhaustive analysis of this type applied to *P. aeruginosa*. Mononucleotide G:C SSRs are under-represented in *P. aeruginosa*, similarly to previous observations for *E. coli* and other bacteria [4], [5], [11], [12]. This phenomenon was much more prominent in coding regions than in non-coding regions of the genome (Figure 1 and S1). Although an intrinsic replicative instability of G:C SSRs could partially account for their under-representation, it is clear that G:C runs in *P. aeruginosa* are strongly counterselected in the coding DNA. This finding suggests that the G:C SSRs that are maintained in the coding regions are of functional/evolutionary relevance because they make certain genes more prone to mutation than others.

### SSRs constitute a major pathway for adaptive mutagenesis during CF chronic infection

**Figure 2:**
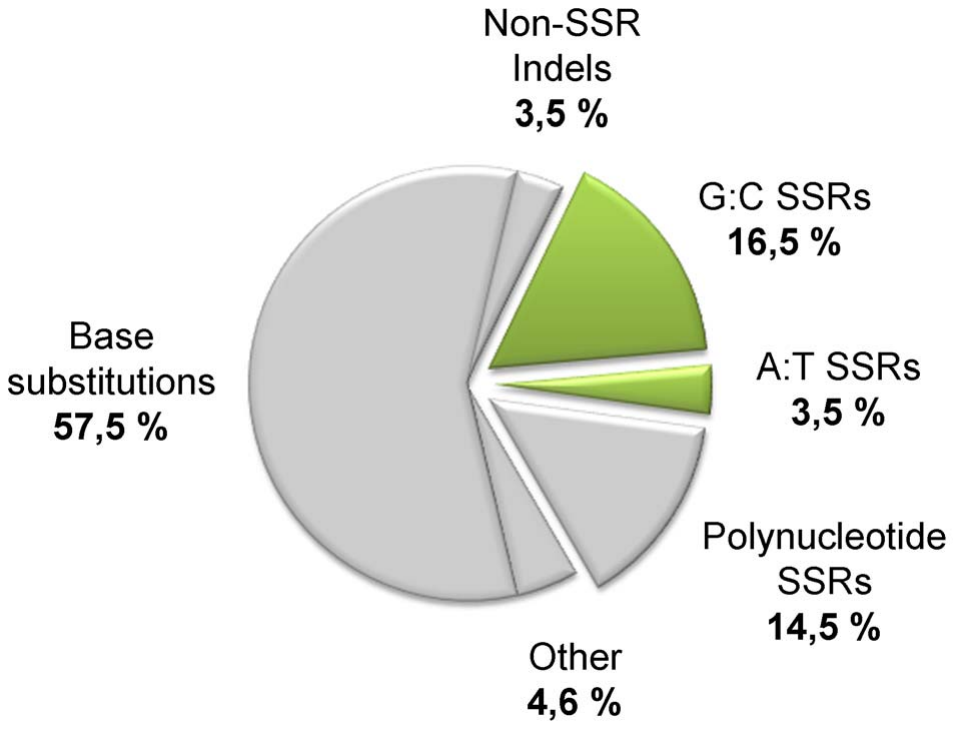
Genetic changes in *P. aeruginosa* PACS2. The pie-chart shows the distribution of 85 independent mutational events that occurred in the genome of *P. aeruginosa* strain PACS2 during the process of chronic infection, as reported by Smith *et al*. [1]. Percentages are shown of base substitutions and of indels falling inside the SSRs or outside (referred to as Non-SSR indels). Large indels (>100 bp) or insertion elements are indicated as “other” mutations. Mutations within SSRs, either mononucleotide or polynucleotide (tandem repetition of short DNA motives), are shown as detached slices. Slices corresponding to mononucleotide G:C and A:T SSRs are shown in green.

In the context of CF chronic infection, the above findings posed the question whether mononucleotide SSRs play a role in genetic adaptation of *P. aeruginosa* to the airways of CF patients. To elucidate the role of SSRs in an *in vivo* context, we performed a meta-analysis of published data from whole-genome sequencing of two *P. aeruginosa* CF isolates from the same patient that were obtained 90 months apart [1]. This 2006 study revealed the genetic changes that made possible the long-term adaptation of this *P. aeruginosa* strain (referred to as PACS2) to the CF lung environment. We used the data from the 2006 study to evaluate the involvement of mononucleotide SSRs in this adaptive process by analyzing 85 independent mutational events (mutations m1-m68 and s1-s17) that occurred in the coding sequences of 60 genes and 9 intergenic regions [1]. More than half of these mutations consisted of some type of base substitution (Figure 2). The other large category of alterations was the insertion/deletion ("indel") mutations, the majority of which (91%) occurred in some type of SSR through the addition or deletion of one or more repeating units of the SSR. The indel mutations found in SSRs most frequently occurred in mononucleotide G:C SSRs (Figure 2).

**Figure 3:**
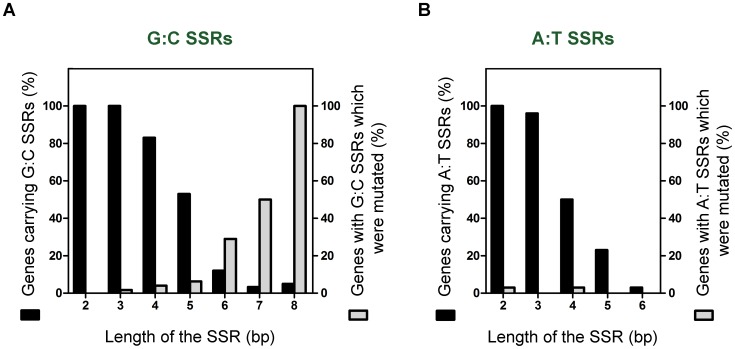
Involvement of mononucleotide SSRs in genes involved in *P. aeruginosa* adaptation during CF chronic infection. The bar graphs show (i) the percentages of the 60 genes mutated during CF lung chronic infection as reported by Smith *et al.* [1] that harbor mononucleotide G:C SSRs (A) and A:T SSRs (B), relative to the length of the SSR (black bars); (ii) the percentages of those genes carrying mononucleotide SSRs that were mutated during the process of chronic infection, relative to the length of the SSR (gray bars).

Of the 60 genes analyzed, the number of those that carried mononucleotide G:C SSRs decreased exponentially (with a common ratio of ∼5.6) with the corresponding increase in SSR length (Figure 3A). When we considered only those genes harboring G:C SSRs that were mutated during the infection process, the proportion increased notably with the size of the SSR. This finding suggests that even for a heterogeneous group of genes the capacity of G:C SSRs to make genes more prone to mutation was size-dependent. Previous studies on the role of SSRs were restricted to single SSRs involved in the acquisition of specific phenotypes [4], [7], [8], [9], [10]. It was therefore possible to assess the more general impact of SSR mutagenesis only by gathering information from various studies of individual cases. Surprisingly, of the 5 G:C SSRs of length 8 bp that were observed in the whole coding DNA of the PACS2 genome, 3 were present within the 60 genes and were mutated during the CF infection process. The A:T SSRs did not display this phenomenon or any association between SSR length and mutagenesis (Figure 3B).

**Figure 4:**
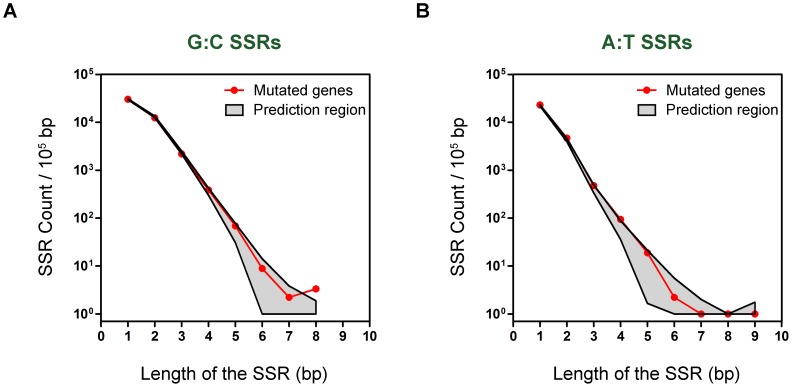
Mononucleotide SSRs in the 60 mutated genes compared with the genome coding region of *P. aeruginosa* PACS2. The plots show the numbers of mononucleotide G:C SSRs (A) and A:T SSRs (B) (normalized to SSR count every 10^5^ bp) in the pooled coding sequence of the 60 genes mutated during the process of chronic infection (red curve) and in the 99% prediction region (grey area) estimated by re-sampling 60 genes at random, with reposition, from the whole coding genome of *P. aeruginosa* PACS2. The sampling was repeated 50000 times to obtain a good estimation of empirical distributions of SSR counts. From these distributions the extreme 0.5% and 99.5% quantiles were estimated to build the 99% prediction intervals for each length of SSR. Points of the curve which fall outside of the prediction area were considered to be statistically significant (p = 0.01).

As another indicator of the functional relevance of SSRs during CF chronic infection, we scored the content of G:C and A:T SSRs in a 99% prediction region, for SSRs of length 1 to 9 bp, that was estimated by a re-sampling technique. A set of 50000 samples containing 60 genes each (the number of genes in the pool of mutated genes) was randomly taken from the whole coding genome of *P. aeruginosa* PACS2 (see Materials and Methods), thereby providing a good estimation of empirical distributions of mononucleotide SSR counts. We determined whether the frequencies of the G:C and A:T SSRs of every length in the pool of 60 mutated genes fell inside or outside the prediction region generated by the 50000 random samples. Among the 60 mutated genes, the frequencies of G:C SSRs with length ≤7 bp fell within the area of the prediction region (Figure 4A). The frequency of G:C SSRs with length 8 bp in the pooled mutated genes was much higher than the value for the predicted interval (p = 0.01). In contrast, the frequencies of A:T SSRs in the prediction region vs. the 60-gene pooled sequences were not significantly different (Figure 4B). These findings indicate that long G:C SSRs were functionally relevant during the genetic adaptation process of *P. aeruginosa* because they made genes more prone to mutation.

In summary, in the environment of CF chronic lung infection, it appears that mononucleotide G:C SSRs constitute length-dependent hotspots for mutagenesis in various genes that are involved in the genetic adaptation process of *P. aeruginosa*.

### Mutagenesis in mononucleotide G:C SSRs, but not A:T SSRs, is synergistically enhanced by stable hypermutability during CF chronic lung infection

The genetic adaptation of *P. aeruginosa* to the airways of CF patients is based primarily on mutational events. The process of genetic adaptation has been suggested by Mena *et al.* to be “catalyzed” by MRS-deficient mutators through an increase in the acquisition of adaptive mutations [9]. These authors observed a tendency toward an increased proportion of small indels but concluded that the incremental effect of hypermutability was not biased toward any specific gene or any particular type of mutation. They did not specifically examine the effect of hypermutability on mononucleotide SSRs. In our previous studies, we used an MRS-deficient mutator strain of *P. aeruginosa* to demonstrate *in vitro* that the frequency and spectrum of mutagenesis of the *mucA* gene was dependent on a G:C SSR of length 5 bp [7], [8]. This finding poses the question of whether hypermutability resulting from MRS deficiency exerts a wide effect on the whole population of genomic G:C SSRs in the context of CF chronic lung infection.

**Figure 5:**
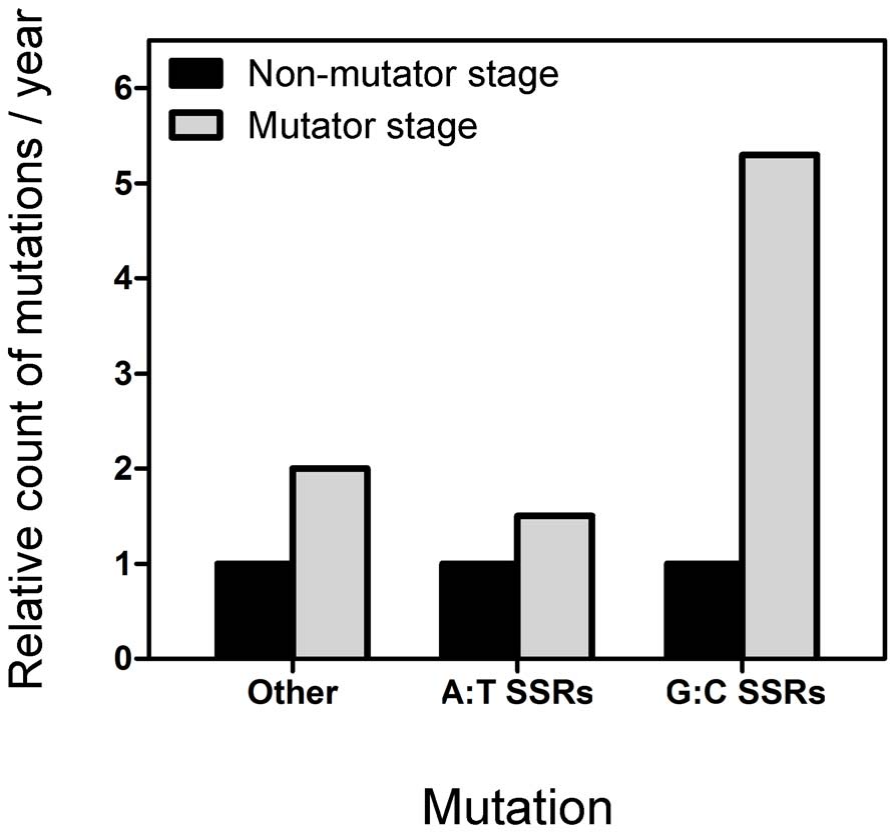
Genome-wide effect of MRS-deficient hypermutability on the mutagenesis of mononucleotide SSRs. The bars show the fold increase in the mutations per year that occurred in mononucleotide G:C SSRs and A:T SSRs, and in other types of mutation after *P. aeruginosa* PACS2 became mutator**.**

To study the specific *in vivo* effect of hypermutability on mononucleotide SSRs, we followed a strategy similar to that of Mena *et al*. [9]. These authors examined the association of hypermutability with the 68 mutations (m1 to m68) that were found to be accumulated in 35 isolates of *P. aeruginosa* strain PACS2 collected over a period of 90 months of infection, as described previously by Smith *et al.* [1]. The isolates collected after 54 months of infection were found to be MRS-deficient mutators. We were thus able to perform a comparison between (i) the mutations in mononucleotide G:C and A:T SSRs that occurred during the first 54 months of infection, when the strain was non-mutator (isolates 1 to 31) vs. (ii) the mutations that accumulated in mononucleotide SSRs during the subsequent 36-month period when the strain became mutator (isolates 32 to 35). We then compared these findings with the general incremental effect of hypermutability on other types of mutations. During the first 54 months of infection, 26 mutations were accumulated (m1 to m26). Of these 26 mutations, 2 (7.7% of the total) were within G:C SSRs and 1 (3.8%) was within A:T SSRs. During the subsequent 36-month period when the strain became mutator, 42 new mutations were observed, of which 7 (16.7%) occurred within G:C SSRs and 1 (2.4%) within A:T SSRs. Thus, when the strain was non-mutator, the rate of accumulation of mutations per year was 0.4 for mononucleotide G:C SSRs, 0.2 for A:T SSRs, and 5.1 for other types of mutation. During the mutator period, the corresponding rate was 2.3 for G:C SSRs, 0.3 for A:T SSRs, and 11.3 for other mutations. Thus, stable hypermutability resulted in a 5.3-fold increase in the rate of accumulation of mutations for G:C SSRs. This increase was notably greater than that for A:T SSRs (1.5-fold) and for other mutations (2.2-fold) (Figure 5).

Smith *et al*. [1] analyzed a set of 90 CF isolates collected from 29 patients (patients 2 to 30) with chronic *P. aeruginosa* infections. These authors sequenced the coding and regulatory regions of 34 genes of the isolates and observed 155 mutations distributed in 22 genes and 9 additional mutations in the regulatory regions. Fifteen of the 90 isolates were shown to be MRS-deficient mutators in a subsequent study [9]. Thus, of the 164 recorded mutations, 66 were first observed in isolates that were mutators. These findings posed the question whether an association between hypermutability and mutagenesis in G:C or A:T SSRs existed in this set of mutations. We found that of the 66 mutations in mutator strains, 7 (10.6%) corresponded to mutations in mononucleotide G:C SSRs. This value was significantly higher (p =  0.031) than those for the non-mutator isolates, which had 98 mutations of which only 2 (2.0%) occurred within G:C SSRs. A similar analysis of A:T SSRs showed only 1 mutation (1.5%) in a mutator strain. This value was not statistically different (p =  0.402) from that for non-mutators, in which no such mutations were observed. These results are consistent with those for patient 1 as above.

In order to extend these observations, we performed a new analysis by using recent data obtained in our lab from the whole genome sequencing of 27 MRS-deficient *P. aeruginosa* isolates from two different CF patients (Feliziani *et al.*, unpublished). This analysis allowed the detection of highly mutated G:C SSRs that could therefore play a relevant role in *P. aeruginosa* adaptation to the CF lung. We chose three of the most frequently mutated G:C SSRs found in the 27 genomes (Table S1) as candidates for further analyses. Two of these G:C SSRs, a 7- and a 6-bp-long SSR, are harbored in genes PA0929 and PA1127 respectively, and the third (8 bp-long) is intergenic, located between genes PA4568 and PA4569. Because these 27 isolates are all MRS-deficient mutators, a comparison between mutators vs. non-mutators was not possible. Furthermore, they belong only to two different clonal lineages (one single linage per patient), thereby being difficult to distinguish whether this high frequency of G:C SSR-mutations corresponds to independent mutational events. In order to circumvent these issues, we further analyzed the chosen G:C SSRs in a new set of 28 isolates obtained from 22 different CF patients with chronic airway infection as described previously [13]. This collection contained several different clones of mutator as well as non-mutator isolates (10 MRS-deficient mutators and 18 non-mutators), thereby allowing to establish whether mutagenesis of these SSRs is a common feature among a clonally diverse set of isolates and whether this phenomenon is associated with hypermutability or not. It’s worth mentioning that previous to this analysis, we confirmed that the candidate G:C SSRs were also fully conserved in reference strains of *P. aeruginosa* such as PAO1, PA14, PACS2, 2192, C3719, DK2 and LESB58 (whose sequences are available online) to ensure that these G:C SSRs and their potential mutations could be detected in the new set of isolates (Table S1).

Interestingly, mutagenesis in two of the three analyzed G:C SSRs showed significant differences between the MRS-deficient mutator and the non-mutator strains (p =  0.010). In this sense, not a single G:C SSR mutation was found in the 18 non-mutator isolates, whereas 40% of the mutator isolates showed indel mutations in the PA0929 G:C SSR, 10% in PA1127 G:C SSR and 40% in the PA4568//PA4569 intergenic G:C SSR (Figure S2).

These findings, taken together, support the hypothesis that becoming a MRS-deficient mutator is a matter of not only the “quantity” but also “quality” of mutations, through the preferential enhancement of mutagenesis in determinate hotspots, such as mononucleotide G:C SSRs. The collected data suggest that in non-mutator strains of *P. aeruginosa* mutagenesis in mononucleotide G:C SSRs exerts a moderate impact on the adaptive process to the airways of patients with chronic infection, whereas under a MRS-deficient mutator background this phenomenon differs in regard to the overall increase in mutation frequency. Paradoxically, such an association was not observed in one of the most relevant cases of G:C SSR mutagenesis in the course of CF chronic lung infection: the -1 deletion in a 5-bp G:C SSR located in the *mucA* gene, which leads to mucoid conversion [13]–[17]. We previously demonstrated an association between this -1 deletion and hypermutability *in vitro* [7], [8]; however, such association was not observed in the CF airway environment *in vivo* [13], [18]. This observation may be explained by the fact that the mucoid phenotype is selected at earlier stages of the infection, usually preceding the onset of MRS-deficient mutator strains [13], [18], [19]. Another possibility is that transient/stress-induced hypermutability also plays a role in G:C SSRs mutagenesis. In *P. aeruginosa* and other bacteria these motifs provide "hot" substrates for the mutagenic activity of error-prone DNA polymerases, such as Pol IV (*dinB*) [7], [20].

## Conclusions

*P. aeruginosa* is the most important pathogen in chronic infections that occur in the airways of CF patients. Data from recent whole-genome sequencing studies show that in order to persist, *P. aeruginosa* undergoes a mutational adaptive process [1] that may be promoted by the emergence of mutator strains [9]. We evaluated the role of mononucleotide G:C and A:T SSRs in this adaptive process. Our results suggest that evolutionary selective pressures shaped the genome coding region of *P. aeruginosa* to prevent the existence of long mononucleotide G:C SSRs. However, long G:C SSRs that are still present are functionally relevant because they make the genes in which they are harbored more prone to mutation. The probability of mutation increased exponentially with the length of the SSR. Although mutagenesis in G:C SSRs may have a moderate impact on the whole “mutagenome” of non-mutator strains, the impact becomes greater in strains that become mutator because of MRS deficiency. We demonstrated a significant association between MRS deficiency and the synergistic increase in mutagenesis of mononucleotide G:C SSRs beyond the general increase in other types of mutations. Considering that the emergence of mutator strains is a frequent outcome in CF patients [6], the virulence-associated genes that have long G:C SSRs are of particular concern under a mutator background.

## Materials and Methods

### Data resources

Annotated sequences of the complete genomes of *P. aeruginosa* strains PAO1 and PACS2 (Accession Numbers NC_002516 and NZ_AAQW01000001, respectively) and of individual genes were downloaded from the National Center for Biotechnology Information (NCBI) (http://www.ncbi.nlm.nih.gov) or from the *Pseudomonas* Genome Database [21] (http://www.pseudomonas.com). The genomic sequence data of the 27 cystic fibrosis isolates is publicly available at the European Nucleotide Archive (Accession: ENA/SRA ERP002379) (http://www.ebi.ac.uk/ena).

### Determination of SSR frequencies and distributions in the genome of *P. aeruginosa*

The Coderet software available from the “Mobyle” site for bioinformatic analysis [22] (http://mobyle.pasteur.fr/cgi-bin/portal.py) was used to extract coding regions from the genome sequence of *P. aeruginosa*. Scoring for SSRs at the genome level or within individual genes was performed using the Simple Sequence Repeats software [10] available at http://www.cmbl.uga.edu/software.html. To determine the expected SSR counts, we used 8 predictive random models of diverse complexities. Of these, 2 were homogeneous models (Bernoulli and first-order Markov) and 6 were heterogeneous models (b-b, b-bp, m1-m1, m1-m1p, m1c, and m1-c1), as described previously [10]. Unlike homogeneous models, heterogeneous models represent the compositional dissimilarities found in the coding vs. non-coding sequences of the genome. These models distinguish coding from non-coding DNA, break down the genomic sequences to generate random simulations for each region separately, and finally reassemble the randomized sequences of both regions. For both the homogeneous and heterogeneous models, the expected SSR count was the average of 10 simulations. The curves from different random models provided a range of expected SSR counts, as described previously [5], [10].

### Determination of prediction intervals for SSR frequencies

To test for the hypothesis that the SSR frequencies are the same within the pool of mutated genes as in the whole coding genome, a 99% prediction interval for each G:C and A:T SSR length was estimated by a re-sampling technique. A set of 50000 samples containing 60 genes each (the number of genes in the pool of mutated genes) was taken at random, with reposition, from the whole coding genome. For each sample the counts of mononucleotide G:C and A:T SSRs of length 1 to 9, normalized to a length of 10^5^ bp, were recorded. From the empirical distributions of (normalized) SSR counts the extreme 0.5% and 99.5% quantiles were estimated to build the 99% prediction intervals for each length of SSR. The procedure is an *ad hoc* algorithm implemented in R 3.0.1 [23].

### Sequence analysis of the PA0929 and PA1127 genes, and the PA4568//PA4569 intergenic region

Genomic DNA of *P. aeruginosa* CF isolates [13] was extracted by using a DNA isolation kit (QIAGEN). Because mononucleotide SSRs are prone to slippage-errors during PCR amplification steps, the reactions were performed with *Pfu* DNA polymerase enzyme (Fermentas) which possesses 3′→5′ exonuclease (proofreading) activity that enables the polymerase to correct nucleotide incorporation errors. PCR amplification and DNA sequencing were carried out using primers 0929-F (5′-GACGATTATCTGCCGAAGCCT-3′) and 0929-R (5′-GGTATTCGGTCGGCGTCA-3′) for PA0929, 1127-F (5′-GCAAGTCCGACCCGAAGTT-3′) and 1127-R (5′-GTCCTTGAGAAAAACCGCCA-3′) for PA1127, and 4568//4569-F (5′-TAAACCTGCTCACCCGACG-3′) and 4568//4569-R (5′-TGTAGCCCAGGGTCTTGCC-3′) for the PA4568//PA4569 intergenic region. PCR amplifications were performed under the following conditions: 8 min at 95°C, 33 cycles of 1 min at 94°C, 1 min 20 sec at 60°C, 2 min at 72°C, and a final 10 min-extension at 72°C. The PCR products were cleaned with a Gel Purification kit (QIAGEN) and both strands were directly sequenced by using the same PCR primers (DNA Sequencing Facility, University of Chicago).

### Statistical analysis

The data were analyzed by the two-sided Fisher’s exact test using GraphPad Instat software. Differences were considered statistically significant for p< 0.05.

## Supporting Information

Figure S1 **Mononucleotide SSRs in the** ***P. aeruginosa*** **PACS2 coding and non-coding genome regions.** The plots show the counts of mononucleotide SSRs in the coding and non-coding regions of the genome (red circles) and in random sequences generated by various predictive models (black symbols), as described in Methods.
(TIF)

Figure S2 **Association between MRS-deficient hypermutability and mutagenesis in three selected G:C SSRs.** Bars indicate the percentage of mutator (gray bars) and non-mutator (black bars) *P. aeruginosa* CF isolates that harbored indel mutations in three selected G:C SSRs, located in genes PA0929, PA1127 and the intergenic region PA4568//PA4569. Statistically significant differences (p< 0.05) are indicated by * (two-sided Fisher’s exact test).
(TIF)

Table S1 **Selected G:C SSRs which were frequently mutated in the genomes of** ***P. aeruginosa*** **isolates from CF patients.**
(TIF)
