# Supplementary figures and images for: Simple Sequence Repeats Together with Mismatch Repair Deficiency Can Bias Mutagenic Pathways in Pseudomonas aeruginosa during Chronic Lung Infection
Source: PLoS One. 2013 Nov 21;8(11):e80514. doi: 10.1371/journal.pone.0080514 (PMC3837008; doi:10.1371/journal.pone.0080514)

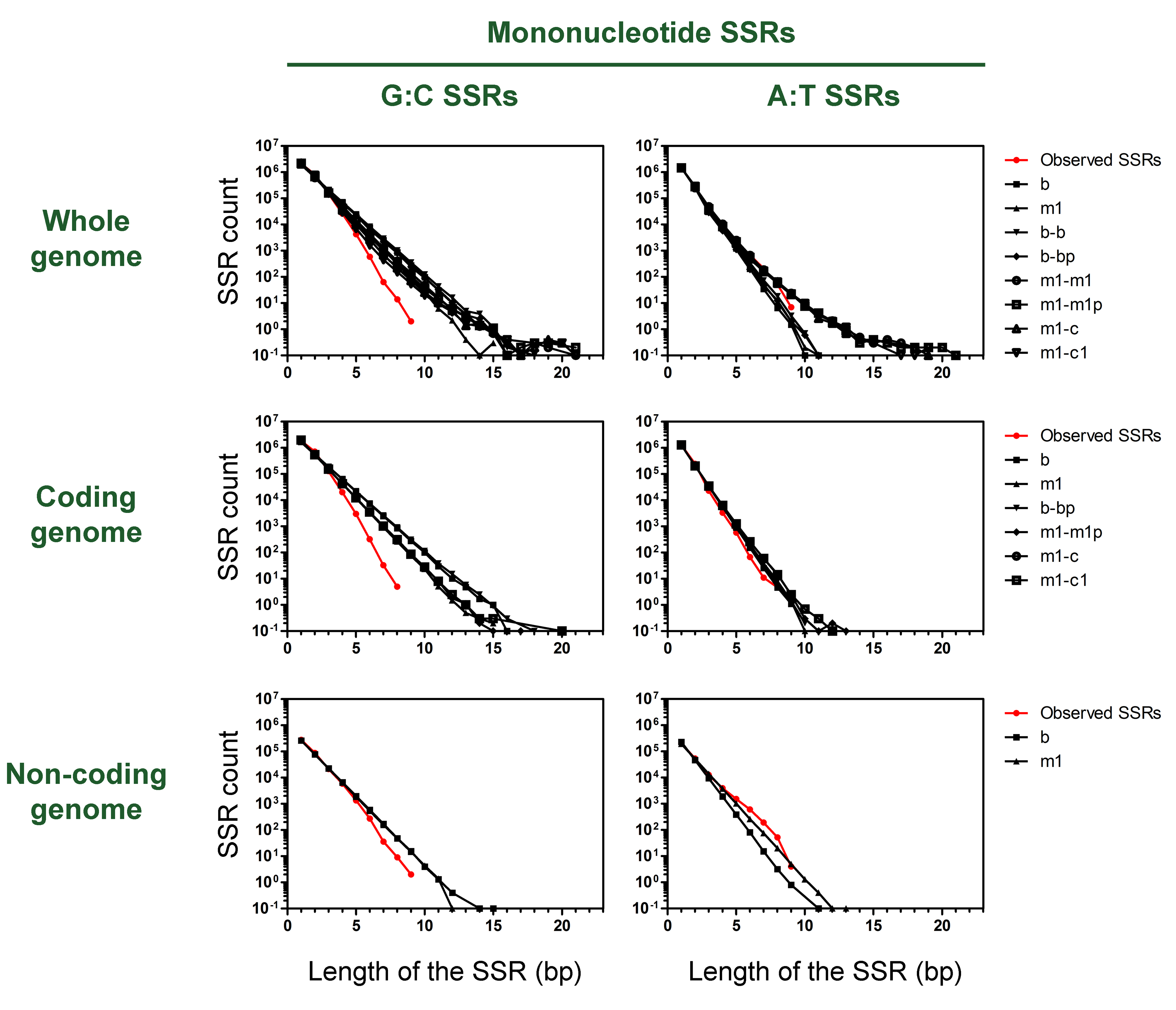

Supplement: Figure S1 — Mononucleotide SSRs in the P. aeruginosa PACS2 coding and non-coding genome regions. The plots show the counts of mononucleotide SSRs in the coding and non-coding regions of the genome (red circles) and in random sequences generated by various predictive models (black symbols), as described in Methods. (TIF) [file pone.0080514.s001.tif]

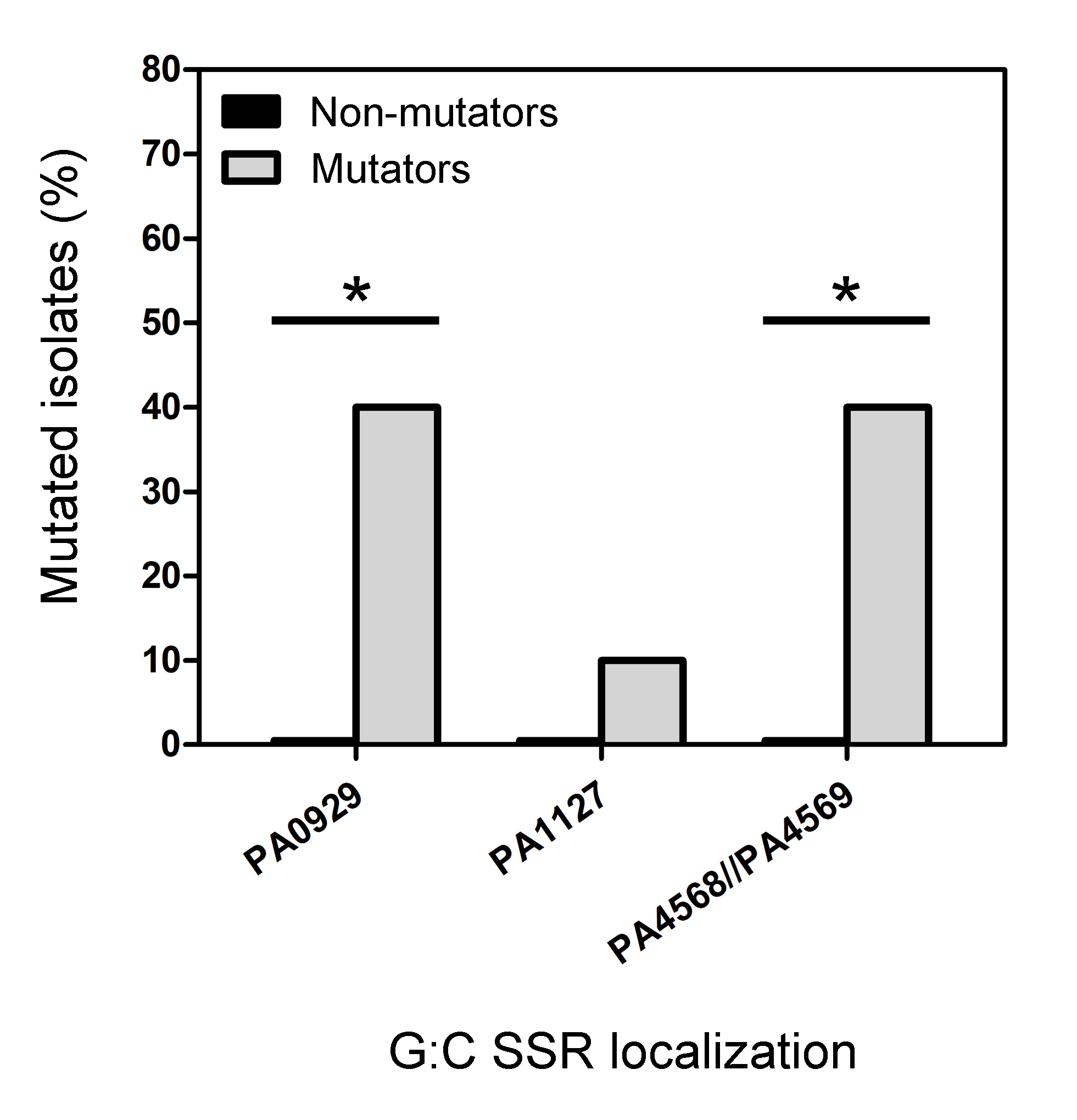

Supplement: Figure S2 — Association between MRS-deficient hypermutability and mutagenesis in three selected G:C SSRs. Bars indicate the percentage of mutator (gray bars) and non-mutator (black bars) P. aeruginosa CF isolates that harbored indel mutations in three selected G:C SSRs, located in genes PA0929, PA1127 and the intergenic region PA4568//PA4569. Statistically significant differences (p< 0.05) are indicated by * (two-sided Fisher’s exact test). (TIF) [file pone.0080514.s002.tif]

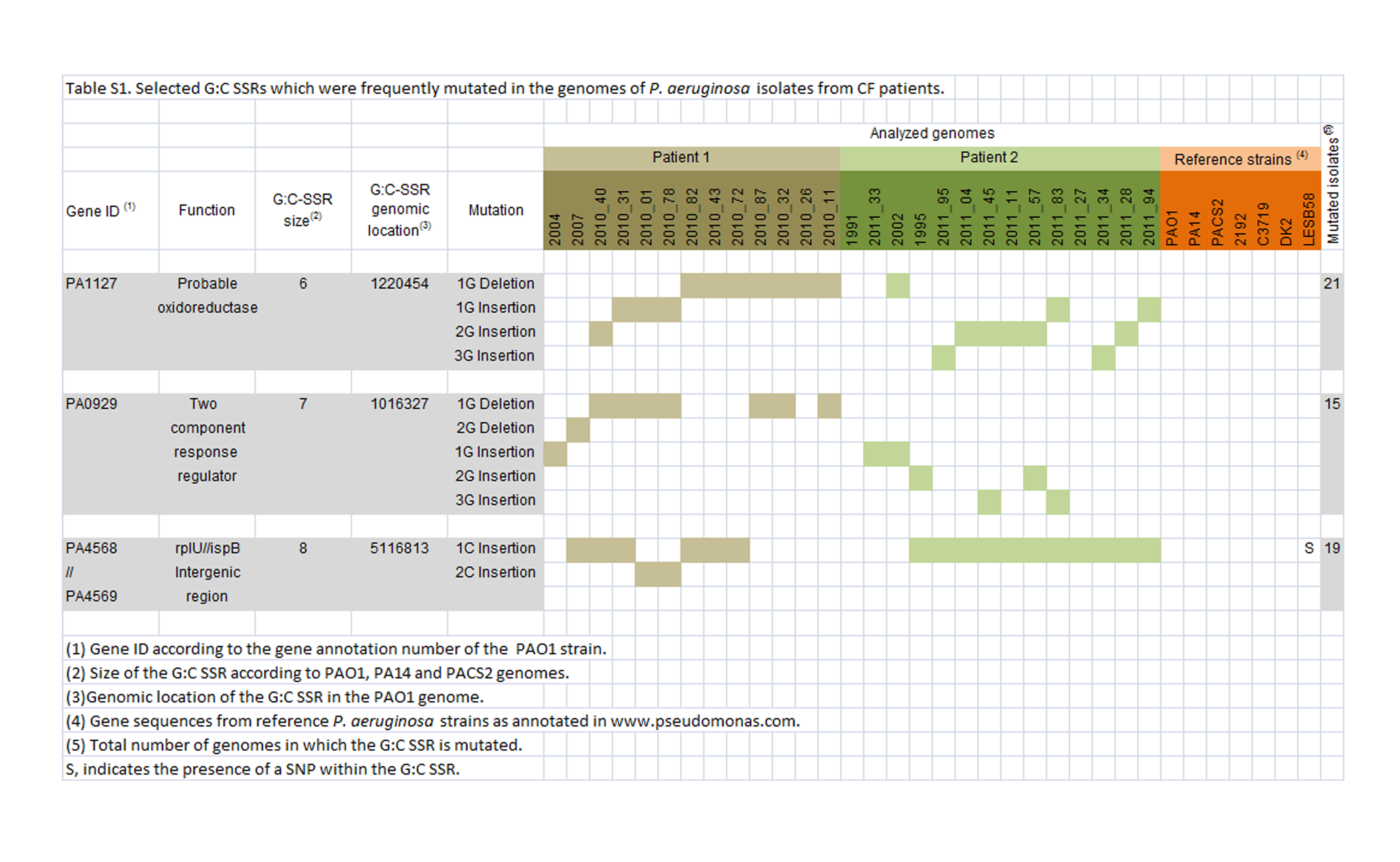

Supplement: Table S1 — Selected G:C SSRs which were frequently mutated in the genomes of P. aeruginosa isolates from CF patients. (TIF) [file pone.0080514.s003.tif]
